# Efficacy and Safety of Dalpiciclib in HR-Positive Advanced Breast Cancer: A Two-Center Retrospective Study

**DOI:** 10.3390/cancers18061025

**Published:** 2026-03-22

**Authors:** Jingjing Li, Zhiqiang Zong, Didi Zhu, Xiaojun Xu, Yunwen Yan, Jia Li, Fanfan Li, Jiqing Hao

**Affiliations:** 1 Department of Oncology, The First Affiliated Hospital of Anhui Medical University, No. 218 Jixi Road, Hefei 230022, China; 2 Department of Oncology, The Second Affiliated Hospital of Anhui Medical University, No. 678 Furong Road, Hefei 230601, China; 3 Department of Breast Surgery, The First Affiliated Hospital of Anhui Medical University, No. 218 Jixi Road, Hefei 230022, China; 4 Department of Breast Surgery, The Second Affiliated Hospital of Anhui Medical University, No. 678 Furong Road, Hefei 230601, China

**Keywords:** Dalpiciclib, HR-positive breast cancer, CDK4/6 inhibitor, endocrine therapy, progression-free survival

## Abstract

**Background**: This study aims to evaluate the real-world efficacy and safety of dalpiciclib in patients with hormone receptor-positive (HR+) advanced breast cancer and explore the impact of different clinical characteristics on treatment outcomes. **Methods**: This was a two-center, retrospective cohort study involving 76 patients treated with dalpiciclib between January 2022 and June 2024 at two affiliated hospitals of Anhui Medical University in China. Data on progression-free survival (PFS), adverse events, and key clinical factors were collected and analyzed. Kaplan–Meier estimates were used for statistical analysis. **Results**: The median PFS (mPFS) for the entire cohort was 12.00 months (95% CI: 10.09–13.91 months). Patients receiving dalpiciclib as first-line therapy had significantly better outcomes (mPFS: 17.00 months, 95% CI: 9.19–24.81 months) than those receiving later-line therapy (*p* < 0.001). Patients with prior exposure to cyclin-dependent kinase 4/6 inhibitors (CDK4/6i) and those with endocrine resistance had poorer outcomes. Multivariate Cox proportional hazards regression analysis confirmed that earlier treatment line (HR for second-line vs. first-line: 3.89, *p* = 0.015; HR for third-line or later vs. first-line: 5.56, *p* = 0.006) and prior CDK4/6i treatment (HR = 3.42, *p* = 0.040) were independent predictors of PFS. The most common adverse events were hematologic toxicities, including leukopenia (76.6%) and neutropenia (72.4%), mostly grade 1–2. No febrile neutropenia cases were reported, indicating a manageable safety profile. **Conclusions**: Dalpiciclib combined with endocrine therapy is associated with favorable efficacy and safety in real-world settings, with early-line treatment and lower tumor proliferative activity associated with better outcomes. While findings suggest potential for clinical application, further large-scale prospective studies are needed to validate its effectiveness in different patient subgroups and optimize treatment strategies.

## Simple Summary

This real-world study evaluated the efficacy and safety of dalpiciclib, a cyclin-dependent kinase 4/6 inhibitor (CDK4/6i), in 76 patients with hormone receptor-positive advanced breast cancer. Our findings demonstrate that dalpiciclib combined with endocrine therapy achieves a median progression-free survival of 12 months, with significantly better outcomes when used as first-line treatment (17 months) compared to later-line therapy (10 months for second-line, 4 months for third-line or later). Multivariate analysis identified the treatment line of dalpiciclib and prior chemotherapy as independent predictors of survival outcomes. The safety profile was manageable, with hematologic toxicities being most common but rarely leading to treatment discontinuation. These real-world results suggest that dalpiciclib is an effective treatment option, particularly when introduced early in the treatment course, and highlight the importance of considering treatment sequencing in clinical decision-making.

## 1. Introduction

According to the latest research data published by the World Health Organization in 2024, breast cancer ranks first in both incidence and mortality among women’s cancers, posing a severe threat to women’s physical and mental health [1]. Among all breast cancer patients, hormone receptor-positive (HR+) and human epidermal growth factor receptor 2-negative (HER2-negative, HER2−) breast cancer accounts for approximately 75%. Endocrine therapy (ET), with its confirmed efficacy and convenient administration, has become the first recommended treatment strategy in clinical practice [2,3]. However, some patients experience decreased efficacy due to primary or secondary resistance during treatment, leading to therapeutic failure [4].

In the early 1970s, the introduction of tamoxifen marked the beginning of oral endocrine therapy for breast cancer. With advances in science and technology, endocrine therapy drugs have continuously been updated and iterated but still fail to meet clinical needs fully. The era of targeted therapy has opened a new chapter for endocrine therapy in HR+ breast cancer. Clinical studies have confirmed that more than half of breast cancer patients overexpress cyclin D, and most of them are ER+ breast cancer patients [5]. Cyclin D directly acts on cyclin-dependent kinase 4/6 (CDK4 and CDK6), which play an important role in cell cycle transitions. Among them, CDK4/6 is a key regulator in transitioning the cell cycle from the G1 phase to the S phase [6]. CDK4/6 can bind to cyclin D to form active complexes that phosphorylate retinoblastoma protein (Rb), releasing E2F transcription factors from the Rb-E2F complex, allowing cells to enter the S phase and initiate DNA replication [7]. In tumor cells, CDK4/6 is abnormally active, causing excessive phosphorylation of Rb, promoting cell cycle progression, and ultimately leading to uncontrolled cell proliferation [8]. CDK4/6 inhibitors (CDK4/6i) can efficiently and precisely inhibit the activity of CDK4 and CDK6 kinases in breast cancer cells, suppressing tumor cell proliferation to exert antitumor effects [9].

Currently, four CDK4/6i—palbociclib, abemaciclib, dalpiciclib, and ribociclib—have been approved in China. Among them, dalpiciclib is the first CDK4/6i independently developed in China, demonstrating good efficacy and safety in treating HR+/HER2− breast cancer [10,11]. In the NCT02684266 [12] and CTR20230830 [13] studies, a recommended dose of 150 mg QD was explored, laying the foundation for large-scale clinical trials in HR+/HER2− breast cancer. In December 2021, dalpiciclib was approved for use with fulvestrant in the treatment of HR+/HER2− recurrent or metastatic breast cancer after prior endocrine therapy. In June 2023, its indications were further expanded for initial treatment of HR+/HER2− locally advanced or metastatic breast cancer in combination with aromatase inhibitors (AI).

While the DAWNA-1 and DAWNA-2 trials established dalpiciclib’s efficacy, several knowledge gaps persist regarding its real-world application. Specifically, it remains unclear whether trial outcomes are reproducible in heterogeneous routine-practice populations. Furthermore, the impact of treatment sequencing (especially post-CDK4/6i exposure) and endocrine sensitivity on dalpiciclib’s performance has not been systematically evaluated in real-world settings. Lastly, real-world safety profiles may diverge from controlled trial data due to diverse patient characteristics and monitoring frequencies. To address these gaps, this two-center retrospective study aims to: (1) evaluate the real-world progression-free survival (PFS) of dalpiciclib plus endocrine therapy in HR+ advanced breast cancer; (2) assess its safety profile in clinical practice; and (3) identify clinical factors associated with outcomes, such as treatment line, prior CDK4/6i use, and endocrine sensitivity. This study provides clinical evidence that complements trial data and informs decision-making in everyday practice.

## 2. Materials and Methods

### 2.1. Study Design and Population

**Figure 1:**
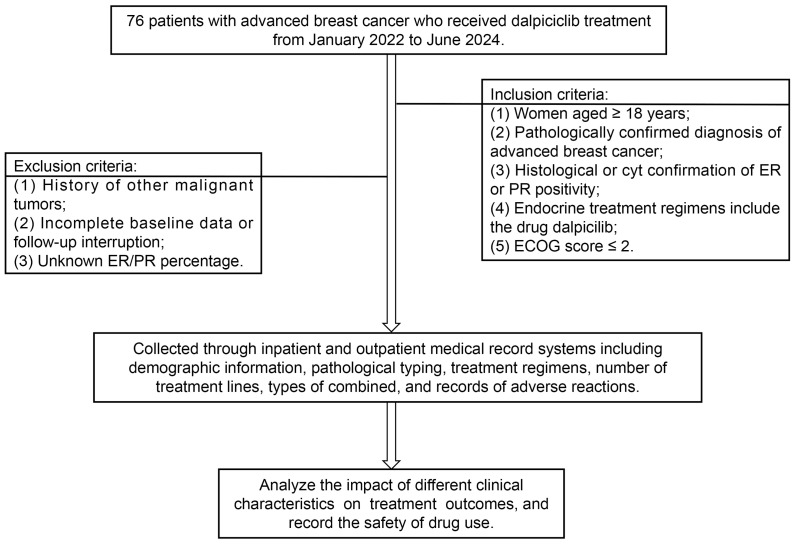
Patient Selection and Data Collection Flowchart for the Study of Dalpiciclib in Advanced Breast Cancer. This flowchart outlines the screening and selection process of the 76 patients included in this study. It summarizes the inclusion and exclusion criteria to ensure a transparent cohort definition for this retrospective analysis.

This study is a two-center, retrospective cohort study that collected clinical data from 76 HR+ advanced breast cancer patients treated with dalpiciclib between January 2022 and June 2024 at the First and Second Affiliated Hospitals of Anhui Medical University (Figure 1). This study was approved by the relevant ethics committees, and all patients signed informed consent forms.

### 2.2. Inclusion Criteria

Patients were eligible for inclusion if they met all of the following criteria: (1) female sex and age ≥ 18 years; (2) pathologically confirmed diagnosis of advanced breast cancer (locally advanced not amenable to curative therapy or metastatic disease); (3) histologically or cytologically confirmed estrogen receptor (ER) or progesterone receptor (PR) positivity, defined as ≥1% positive tumor cells by immunohistochemistry (IHC); (4) treatment with an endocrine therapy regimen that included dalpiciclib at any line of therapy; and (5) Eastern Cooperative Oncology Group (ECOG) performance status ≤2, indicating that patients were ambulatory and capable of self-care (ECOG 0–1) or ambulatory >50% of waking hours and capable of self-care but unable to work (ECOG 2).

### 2.3. Exclusion Criteria

Patients were excluded from the study if they met any of the following criteria: (1) history of other malignancies (except adequately treated non-melanoma skin cancer or carcinoma in situ of the cervix); (2) incomplete baseline data or loss to follow-up during the study period; or (3) unknown ER/PR percentage precluding confirmation of hormone receptor status.

### 2.4. Sampling Method

A consecutive sampling approach was used: all patients with HR+ advanced breast cancer who received dalpiciclib-based therapy at the participating institutions during the study period were screened for eligibility. All eligible patients during the recruitment period (January 2022 to June 2024) were included, constituting a consecutive case series.

### 2.5. Data Collection and Definition

Clinical data were collected through inpatient and outpatient medical record systems, including demographic information, pathological subtypes, treatment regimens, treatment lines, types of combination drugs, and adverse reaction records. The study focused on the following key variables:

PFS: Defined as the time from initiation of dalpiciclib treatment to disease progression or death from any cause, whichever occurred first. Disease progression was assessed by investigators using RECIST version 1.1 criteria based on imaging evaluations (computed tomography [CT] or magnetic resonance imaging [MRI]) performed approximately every 8–12 weeks, or more frequently if clinically indicated. In cases where imaging was not available (e.g., patients with only bone metastases or those unable to undergo regular imaging), clinical progression was determined based on worsening symptoms, deterioration in performance status, or need for palliative radiotherapy, as documented in medical records. Patients without documented progression were censored at the date of last follow-up.

Adverse Events: Graded according to the Common Terminology Criteria for Adverse Events (CTCAE) version 4.03 (grades 0–5).

Hormone Receptor Status: ER and PR expression levels were detected using IHC, with positivity defined as ≥1% nuclear staining.

HER2 Status: Assessed using IHC combined with fluorescence in situ hybridization (FISH) [14].

Endocrine Sensitivity: Endocrine sensitivity is defined as disease recurrence >12 months after adjuvant endocrine therapy, clinical benefit ≥6 months from first-line endocrine therapy, or no prior endocrine treatment; Endocrine resistance is defined as disease recurrence during adjuvant endocrine therapy or within 12 months after its completion, or progression within 6 months of first-line endocrine therapy for advanced disease [15].

### 2.6. Statistical Analysis

All data were analyzed using SPSS Statistics 26.0 (IBM, Armonk, NY, USA). Continuous variables were expressed as mean ± standard deviation ($\bar{x}$ ± s) or median (range), while categorical variables were expressed as frequency and percentage. Comparisons between groups were performed using the chi-square test or Fisher’s exact test. Kaplan–Meier survival curves were plotted by ggplot2 package in R 4.2.1 (R Foundation for Statistical Computing, Vienna, Austria), and the log-rank test was used for intergroup comparisons. Multivariate Cox proportional hazards regression analysis was performed using the enter method to identify independent predictors of PFS. Variables included in the model were those with clinical relevance or a *p*-value < 0.10 in the univariate analysis, with additional adjustment for age, HR status, HER2 status, and Ki-67 to control for potential confounding. A *p*-value of <0.05 was considered statistically significant.

## 3. Results

### 3.1. Clinical and Pathological Characteristics of Patients

This study included a total of 76 patients with HR+ advanced breast cancer, exhibiting a certain degree of diversity in clinical and pathological characteristics. The median age of the patients was 54 years, ranging from 26 to 78 years. Postmenopausal patients accounted for 53.9% (44/76), while premenopausal patients made up 42.1% (32/76). The vast majority of patients (89.5%, 68/76) had an ECOG score of 0–1, indicating a relatively good physical condition. The Ki-67 proliferation index showed that 46.1% of patients had a Ki-67 index <30%, while 53.9% had a Ki-67 index ≥30%. This suggests a certain degree of tumor proliferation heterogeneity within the study cohort, which may influence treatment response and prognosis. More details were presented in Table 1.

To further explore heterogeneity across treatment lines, we compared baseline characteristics among patients receiving first-line, second-line, and third-line or later dalpiciclib therapy. As shown in Supplementary Table S1, patients in later lines had significantly higher rates of prior chemotherapy exposure (*p* = 0.003), prior CDK4/6i use (*p* = 0.026), and endocrine resistance (*p* < 0.001), and were more likely to receive fulvestrant rather than AI (*p* < 0.001). These differences underscore the need for multivariate adjustment in subsequent analyses.

### 3.2. Treatment

In this study, all patients received treatment with dalpiciclib in combination with endocrine therapy. The mPFS for the entire patient cohort was 12.00 months (95% CI: 10.09–13.91 months). Upon further analysis of the impact of different clinical characteristics on treatment outcomes, the following factors were found to significantly influence PFS (*p* < 0.05).

**Figure 2:**
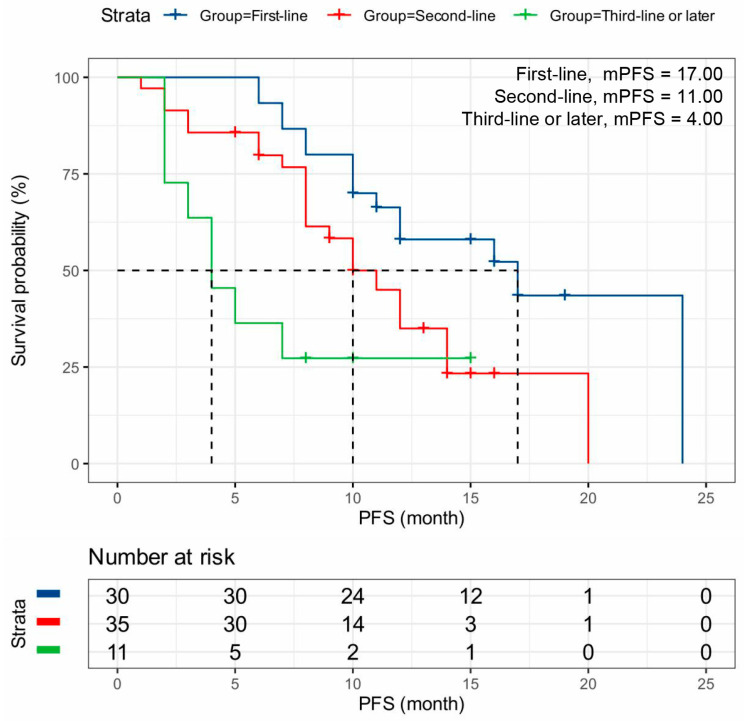
Kaplan–Meier Plots for Dalpiciclib Efficacy in Different Treatment Lines. Kaplan–Meier survival curves showing PFS stratified by line of dalpiciclib therapy: first-line (*n* = 30), second-line (*n* = 35), and third-line or later (*n* = 11). The mPFS was 17.0, 10.0, and 4.0 months, respectively (log-rank *p* < 0.001). Earlier treatment initiation was associated with significantly longer PFS. Dashed horizontal and vertical lines indicate the 50% survival probability and the corresponding mPFS. Abbreviations: PFS, progression-free survival; mPFS, median progression-free survival.

**Figure 3:**
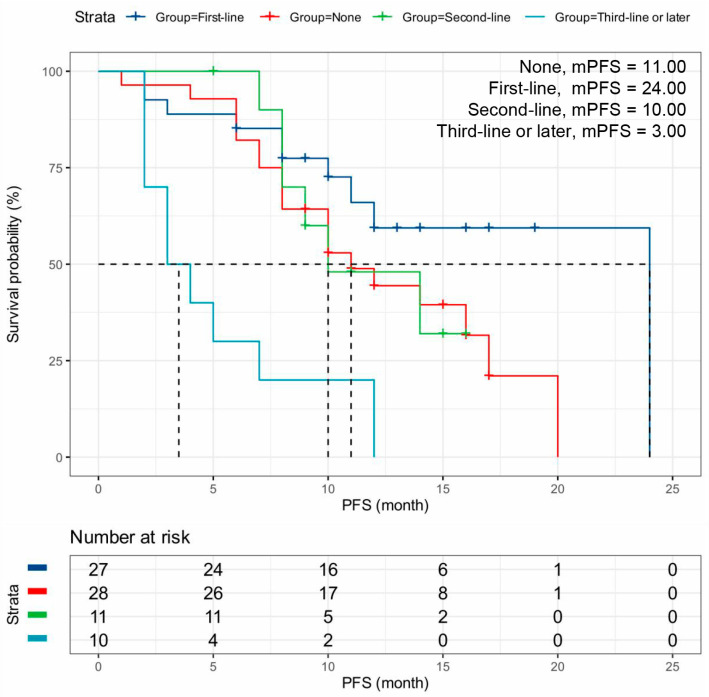
Kaplan–Meier Plot for Prior Lines of Chemotherapy Analysis. Kaplan–Meier curves for PFS stratified by number of prior chemotherapy lines: none (*n* = 27), first-line (*n* = 11), second-line (*n* = 10), and third-line or later (*n* = 28). Median PFS was 11.00, 24.00, 10.0, and 3.0 months, respectively (log-rank *p* < 0.001). Fewer prior chemotherapy lines were associated with significantly longer PFS following dalpiciclib treatment. Dashed horizontal and vertical lines indicate the 50% survival probability and the corresponding mPFS. Abbreviations: PFS, progression-free survival; mPFS, median progression-free survival.

**Figure 4:**
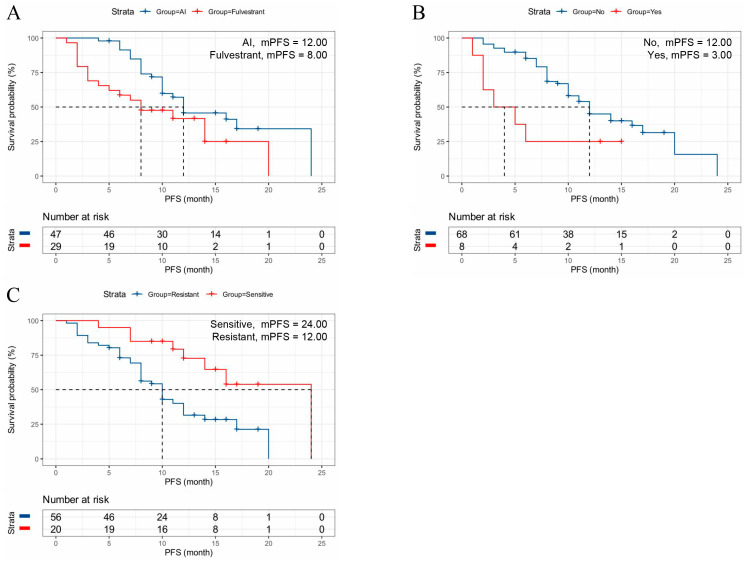
Subgroup Analysis of PFS in Patients Treated with Dalpiciclib. (**A**) Combined endocrine therapy type (AI vs. Fulvestrant); (**B**) Impact of prior CDK4/6i treatment; (**C**) Endocrine sensitivity status (Sensitive vs. Resistant). All survival outcomes were analyzed using the Kaplan–Meier method and compared with the log-rank test. These panels highlight that dalpiciclib efficacy is significantly influenced by prior CDK4/6i exposure (*p* = 0.013) and baseline endocrine sensitivity (*p* = 0.004), while the choice of AI vs. fulvestrant often reflects the patient’s underlying clinical setting. Dashed horizontal and vertical lines indicate the 50% survival probability and the corresponding mPFS. Abbreviations: PFS, progression-free survival; mPFS, median progression-free survival; AI, aromatase inhibitors; CDK4/6i, cyclin-dependent kinase 4/6 inhibitors.

Patients receiving dalpiciclib as first-line therapy were associated with significantly better outcomes (mPFS: 17.00 months, 95% CI: 12.05–19.96 months) compared to those receiving it as later-line therapy (*p* < 0.001) (Table 2, Figure 2). In this study, advanced breast cancer patients with First-line chemotherapy had higher mPFS than those with other lines of chemotherapy (*p* < 0.001) (Table 2, Figure 3). Patients treated with dalpiciclib in combination with AI (mPFS: 12.00 months, 95% CI: 7.16–16.84 months) achieved superior outcomes compared to those treated with dalpiciclib plus fulvestrant (mPFS: 8.00 months, 95% CI: 2.58–13.42 months) (Table 2, Figure 4A). Patients with prior exposure to CDK4/6i had lower mPFS (*p* = 0.013) (Table 2, Figure 4B). Additionally, patients with endocrine resistance (mPFS: 12.00 months, 95% CI: 10.88–14.81 months) exhibited poorer outcomes than those with endocrine sensitivity (mPFS: 24.00 months, *p* = 0.004) (Table 2, Figure 4C).

No significant difference in PFS was observed between patients with <3 distant metastases and those with ≥3 distant metastases (*p* = 0.925) (Table 2). Similarly, the difference in PFS between patients with liver metastases and those without liver metastases did not reach statistical significance (*p* = 0.733) (Table 2). No significant difference in PFS was observed between patients with ER < 50% and those with ER ≥50% (*p* > 0.05) (Table 2).

### 3.3. Cox Regression Analysis

To identify independent predictors of PFS, multivariate Cox proportional hazards regression was performed including variables with clinical relevance or *p* < 0.10 in univariate analysis: line of dalpiciclib therapy, prior lines of chemotherapy, endocrine sensitivity, prior CDK4/6i use, and type of endocrine combination. The model was additionally adjusted for age, HR status, HER2 status, and Ki-67 index.

As shown in Table 3, after adjustment, later lines of dalpiciclib therapy remained independently associated with significantly worse PFS (second-line vs. first-line: HR = 3.89, 95% CI: 1.30–11.61, *p* = 0.015; third-line or later vs. first-line: HR = 5.56, 95% CI: 1.66–21.46, *p* = 0.006). Prior lines of chemotherapy also emerged as independent predictors: compared to no prior chemotherapy, first-line (HR = 0.16, 95% CI: 0.06–0.43, *p* < 0.001) and second-line chemotherapy (HR = 0.26, 95% CI: 0.08–0.86, *p* = 0.026) were associated with improved PFS, whereas third-line or later chemotherapy was not (HR = 1.20, 95% CI: 0.42–3.43, *p* = 0.740). Prior CDK4/6i use remained a significant adverse factor (HR = 3.42, 95% CI: 1.06–11.09, *p* = 0.040). Endocrine resistance and use of fulvestrant (vs. AI) lost statistical significance, suggesting their effects may be mediated by treatment line and prior chemotherapy. Given the modest sample size, the wide confidence intervals for some estimates (e.g., prior CDK4/6i treatment) indicate limited precision; these findings should be considered hypothesis-generating and require validation in larger cohorts.

**Table 3:** Univariate and multivariate Cox regression analysis of factors associated with PFS.

Variable	Univariate Analysis		Multivariate Analysis	
	HR (95% CI)	*p*-Value	HR (95% CI)	*p*-Value
Line of dalpiciclib treatment				
First-line	Reference		Reference	
Second-line	2.10 (1.06–4.17)	0.034	3.89 (1.30–11.61)	0.015
Third-line or later	4.73 (1.92–11.68)	0.001	5.56 (1.66–21.46)	0.006
Prior lines of chemotherapy				
None	Reference		Reference	
First-line	0.49 (0.22–1.08)	0.078	0.16 (0.06–0.43)	< 0.001
Second-line	0.92 (0.37–2.32)	0.863	0.26 (0.08–0.86)	0.026
Third-line or later	3.79 (1.71–8.39)	0.001	1.20 (0.42–3.43)	0.740
Endocrine sensitivity				
Sensitive	Reference		Reference	
Resistant	2.99 (1.33–6.76)	0.008	1.57 (0.63–3.96)	0.337
Prior CDK4/6i treatment				
No	Reference		Reference	
Yes	2.80 (1.17–6.70)	0.021	3.42 (1.06–11.09)	0.040
Dalpiciclib combined with endocrine drugs				
AI	Reference		Reference	
Fulvestrant	1.91 (1.05–3.50)	0.035	0.98 (0.41–2.36)	0.968

Note: multivariate model was additionally adjusted for age, HR status, HER2 status, and Ki-67. Abbreviations: PFS, progression-free survival; HR, hazard ratio; CI, confidence interval; HR status, hormone receptor status; HER2, human epidermal growth factor receptor 2. CDK4/6i, cyclin-dependent kinase 4/6 inhibitors.

### 3.4. Adverse Events

Complete and detailed adverse event records were available for 51 of the 76 patients (67.1%); for the remaining 25 patients, adverse event data were incompletely documented in the medical records, which is an inherent limitation of retrospective studies. This was primarily due to inconsistent documentation during follow-up visits at outside facilities where records could not be obtained, as well as variable documentation practices for non-severe adverse events in routine clinical care.

Among the 51 patients with available safety data, hematologic toxicity was the most common side effect of dalpiciclib treatment, with the incidence of leukopenia being 76.5% and neutropenia being 72.5%. Most of these adverse reactions were manageable grade 1–2 events and did not require discontinuation of treatment. However, for grade 3 or higher adverse events, patients typically recovered through dose adjustments or temporary drug discontinuation. Non-hematologic toxicities were relatively mild, primarily manifesting as abnormal liver function, rash, and elevated uric acid levels, with most being grade 1–2. No cases of febrile neutropenia were reported, further demonstrating the favorable safety profile of dalpiciclib. More details were presented in Table 4.

To assess potential selection bias, we compared baseline characteristics between patients with (*n* = 51) and without (*n* = 25) complete safety data (Supplementary Table S2). Aside from a higher proportion of premenopausal women in the group with complete safety data (*p* = 0.027), no significant differences were observed in age, treatment line, endocrine sensitivity, or other key clinical variables (all *p* > 0.05). This indicates that the subset with available safety data is generally representative of the overall cohort.

### 3.5. Sensitivity Analysis

To assess the robustness of our findings and address potential confounding from heavily pretreated patients, we performed a sensitivity analysis restricted to patients receiving first- or second-line dalpiciclib therapy (*n* = 65), excluding the third-line or later group (*n* = 11). Multivariate Cox regression was conducted with the same covariates as in the primary analysis (line of therapy, endocrine sensitivity, prior CDK4/6i use, type of endocrine combination, adjusted for age, HR status, HER2 status, and Ki-67). As shown in Supplementary Table S3, second-line treatment remained independently associated with significantly poorer PFS compared to first-line treatment (HR = 4.16, *p* = 0.019), consistent with the main analysis. Overall, these results support the robustness of our primary conclusion regarding the independent prognostic value of treatment line.

## 4. Discussion

This study adheres strictly to the principles of real-world research, focusing on analyzing the efficacy of dalpiciclib and its correlation with patient characteristics. The results suggest that dalpiciclib combined with endocrine therapy is associated with significant efficacy in HR+ advanced breast cancer, especially in early treatment settings.

To further support these findings, we performed a multivariate Cox proportional hazards regression analysis. The results confirmed that the line of dalpiciclib treatment is an independent prognostic factor. Specifically, patients receiving dalpiciclib as a later-line therapy faced a significantly higher risk of progression (HR for second-line: 3.89; HR for third-line or later: 5.56) compared to first-line users. Furthermore, prior CDK4/6i exposure was identified as another independent risk factor (HR = 3.42, *p* = 0.040). This robust statistical evidence underscores that while dalpiciclib is effective across multiple settings, its maximum benefit is derived when used earlier in the treatment continuum and in CDK4/6i-naive patients.

In studies on HR+ advanced breast cancer, the DAWNA-1 trial compared the efficacy and safety of dalpiciclib combined with fulvestrant versus placebo combined with fulvestrant in HR+/HER2− patients with advanced breast cancer who had experienced disease progression following prior endocrine therapy. The study reported a median PFS of 15.70 months in the dalpiciclib group versus 7.20 months in the placebo group [16]. Similarly, the DAWNA-2 trial showed a significantly longer PFS in the dalpiciclib combined with AI group compared to the placebo combined with AI group (30.60 months vs. 18.20 months) [17]. The median PFS of 17.0 months for first-line dalpiciclib in our real-world cohort was notably lower than the 30.6 months reported in the DAWNA-2 trial [17]. This discrepancy warrants careful consideration of key differences between trial and real-world populations. First, DAWNA-2 enrolled exclusively treatment-naïve advanced breast cancer patients with strict eligibility criteria, including ECOG score 0–1 and no prior systemic therapy for advanced disease. In contrast, our first-line subgroup included patients who may have received adjuvant chemotherapy within the past 12 months or had subtle comorbidities that would have excluded them from trial participation. Second, our cohort had a higher proportion of patients with aggressive disease features: 53.9% had Ki-67 ≥ 30%. Third, real-world imaging intervals are often less frequent and more variable than protocol-mandated assessments in clinical trials, potentially leading to later detection of progression. Fourth, 11.5% of our patients had prior CDK4/6i exposure-a population explicitly excluded from DAWNA-2. These factors collectively illustrate the well-recognized efficacy-effectiveness gap between randomized controlled trials and real-world practice [18]. Importantly, our findings complement trial data by demonstrating that dalpiciclib remains effective in a broader, more heterogeneous patient population, albeit with attenuated benefit.

At the SABCS conference on 11 December 2024, Professor Fei Ma presented a phase II study exploring the efficacy and safety of dalpiciclib combined with ET in HR+/HER2− advanced breast cancer patients with visceral crisis [18]. Compared to the chemotherapy group, the dalpiciclib combined with ET group achieved a longer median PFS (10.74 months vs. 4.63 months; HR = 0.32, *p* < 0.001). In this study, among 57 patients treated with dalpiciclib combined with AI, the median PFS was 10.95 months (95% CI: 7.71–12.29), whereas for 38 patients treated with dalpiciclib combined with fulvestrant, the median PFS was 8.09 months (95% CI: 4.73–11.23). The results indicate a better prognosis with dalpiciclib combined with AI. Furthermore, univariate analysis using the log-rank test provided support for the benefits of early-line dalpiciclib use, with median PFS values of 10.43 months and 10.67 months for first-line and second-line treatments, respectively, compared to 5.41 months for third-line or later treatments. These findings further support the positive role of dalpiciclib in early-line treatment.

In real-world studies, the combination of AI and dalpiciclib showed a longer mPFS than fulvestrant plus dalpiciclib (12.00 vs. 8.00 months), consistent with findings from Professor Ma Fei’s team at SABCS 2024 [18]. This difference may be due to treatment line and patient characteristics: the AI group mainly included endocrine-sensitive patients receiving first-line therapy, while the fulvestrant group consisted largely of endocrine-resistant, pre-treated patients [19,20,21]. The latter typically present with more aggressive disease and reduced treatment sensitivity. Further randomized controlled trials are needed to validate these observations.

This study found that the mPFS of the first-line chemotherapy subgroup (24 months) was significantly higher than that of the non-chemotherapy subgroup (11 months). A plausible biological rationale may be that DNA damage induced by prior chemotherapy could act synergistically with CDK4/6i-mediated cell cycle arrest (specifically G1-phase blockade that impedes DNA repair machinery), thereby potentiating tumor cell cytotoxicity and/or suppressing proliferative capacity [22,23]. However, it also needs to be interpreted with caution: heterogeneity of chemotherapy regimens and small sample size may all affect the reliability of the results.

Endocrine-resistant patients (mPFS: 12.00 months) had significantly worse outcomes than endocrine-sensitive patients (mPFS: 24.00 months; *p* = 0.004), highlighting the prognostic value of endocrine-sensitive status for dalpiciclib efficacy. This finding aligns with prior evidence that endocrine-resistant tumors often harbor molecular alterations such as RB1 loss or PI3K/AKT/mTOR activation, reducing responsiveness to CDK4/6 inhibition [3,24]. In contrast, endocrine-sensitive patients benefit more from early combination therapy due to preserved estrogen signaling [17]. These results support stratified treatment approaches and the need for novel combinations to overcome resistance [25].

In terms of adverse events, hematologic toxicities were the most commonly observed side effects of dalpiciclib treatment, primarily including neutropenia, leukopenia, anemia, and thrombocytopenia. No cases of febrile neutropenia were recorded, and most patients experienced symptom improvement after dose adjustments or temporary discontinuation. Non-hematologic toxicities were relatively mild, mostly grade 1–2, further supporting the safety profile of dalpiciclib. In China, where the prevalence of hepatitis B virus infection is high and many breast cancer patients have a history of chemotherapy, the risk of liver damage is further exacerbated. The molecular structure of most CDK4/6i includes para-phenylenediamine, which can react with glutathione abundant in the liver to produce a potentially hepatotoxic adduct, known as the glutathione trapping (GSH trapping) effect. Dalpiciclib’s design considers the clinical needs of domestic patients by introducing a piperidine structure that eliminates the risk of glutathione trapping, thus avoiding potential hepatotoxicity. This advantage was also observed in the DAWNA-1 and DAWNA-2 trials, underscoring dalpiciclib’s favorable hepatic safety profile. Overall, dalpiciclib is well-tolerated, and its manageable adverse events lay a solid foundation for long-term use.

This study also raises some new questions. Further research is needed to determine whether dalpiciclib’s efficacy differs significantly across different resistance types. In this study, dalpiciclib was predominantly used in later treatment lines, with some patients having undergone multiple intravenous chemotherapy regimens. These patients often presented with poor physical condition and heavy tumor burden, resulting in poorer survival outcomes for primary and secondary endocrine-resistant patients. Although dalpiciclib has shown significant efficacy in HR+/HER2− patients, its efficacy in HR+/HER2+ patients remains to be further verified, particularly regarding the potential benefits of combination therapy with HER2-targeted agents.

This study has several notable strengths. First, to our knowledge, this is one of the first real-world studies to evaluate the efficacy and safety of dalpiciclib in Chinese patients with HR-positive advanced breast cancer, providing valuable complementary evidence to the pivotal DAWNA trials. Second, our two-center cohort reflects routine clinical practice, offering insights into patient populations often underrepresented in clinical trials, including those with prior CDK4/6i exposure, heavy pretreatment, and aggressive disease features such as visceral crisis. Third, all efficacy assessments were based on investigator-assessed RECIST 1.1 criteria, and data collection was systematically conducted through electronic medical records with centralized review of key variables to ensure data integrity. Fourth, we performed comprehensive statistical analyses, including multivariate Cox regression and sensitivity analyses, to adjust for potential confounders and assess the robustness of our findings.

However, several limitations must be acknowledged. First, the retrospective design introduces inherent selection bias and information bias; although we implemented strict inclusion/exclusion criteria and performed multivariate adjustment, unmeasured confounding cannot be entirely excluded. Second, the modest sample size (*n* = 76) limits statistical power, particularly for subgroup analyses, and precludes more advanced techniques such as propensity score matching. Consequently, estimates for small subgroups (e.g., prior CDK4/6i use, *n* = 8) have wide confidence intervals and should be interpreted with caution. Third, complete adverse event data were available for only 51 patients (67.1%); although we compared baseline characteristics between patients with and without safety data and found no significant differences, potential reporting bias cannot be entirely ruled out. Fourth, the exploratory nature of our subgroup analyses, combined with the absence of multiple testing corrections, means these findings are hypothesis-generating rather than definitive. Fifth, the substantial collinearity between treatment line, prior chemotherapy, endocrine sensitivity, and choice of endocrine partner may have influenced the stability of some multivariate estimates, underscoring the need for validation in larger cohorts. Finally, as an observational study, our findings describe associations and do not establish causality. Despite these limitations, this study provides clinically relevant real-world evidence that complements randomized trial data and generates hypotheses for optimizing dalpiciclib-based treatment strategies. Prospective studies with larger sample sizes are warranted to confirm our findings and to further elucidate mechanisms of resistance.

## 5. Conclusions

In summary, this two-center retrospective study provides real-world evidence that dalpiciclib combined with endocrine therapy is associated with favorable efficacy and a manageable safety profile in patients with HR-positive advanced breast cancer. Multivariate Cox regression analysis identified first-line dalpiciclib treatment and fewer prior lines of chemotherapy as independent predictors of prolonged PFS, while prior CDK4/6i use was independently associated with poorer outcomes. Endocrine resistance and choice of endocrine partner lost statistical significance after multivariate adjustment, suggesting their effects may be mediated by treatment line and prior treatment history. These hypothesis-generating findings underscore the importance of early introduction of dalpiciclib and careful consideration of treatment sequencing in clinical practice. Prospective studies with larger cohorts are warranted to validate these observations and to explore optimal strategies for overcoming resistance.

## Figures and Tables

**Table 1:** Clinical characteristics of 76 patients with HR-positive advanced breast cancer treated with Dalpiciclib.

Characteristics	*n* (%)	Characteristics	*n* (%)
Age (years)		Visceral metastasis	
≥54	41 (53.9)	Yes	30 (39.5)
<54	35 (46.1)	No	46 (60.5)
Menopausal status		Only bone metastases	
Yes	32 (42.1)	Yes	13 (17.1)
No	44 (57.9)	No	63 (82.9)
ECOG score		Liver metastases	
0–1	68 (89.5)	Yes	24 (31.6)
2	8 (10.5)	No	52 (68.4)
Ki-67		Line of Dalpiciclib treatment	
<30%	35 (46.1)	First-line	30 (39.5)
≥30%	41 (53.9)	Second-line	35 (46.1)
HR status		Third-line or later	11 (14.5)
ER + &PR−	12 (15.8)	Dalpiciclib combined with endocrine drugs	
ER + &PR+	64 (84.2)	AI	47 (61.8)
Percentage of ER		Fulvestrant	29 (38.2)
<50%	6 (7.9)	Prior lines of chemotherapy	
≥50%	70 (92.1)	None	28 (36.8)
Percentage of PR		First-line	27 (35.5)
<50%	39 (51.3)	Second-line	11 (14.5)
≥50%	37 (48.7)	Third-line or later	10 (13.2)
HER2 status		Prior CDK4/6i treatment	
Negative	70 (92.1)	Yes	8 (10.5)
Positive	6 (7.9)	No	68 (89.5)
Number of distant metastases		Endocrine sensitivity	
<3	47 (61.8)	Sensitive	20 (26.3)
≥3	29 (38.2)	Resistant	56 (73.7)

Abbreviations: HR, hormone receptor; ER, estrogen receptor; PR, progesterone receptor; ECOG, Eastern Cooperative Oncology Group; AI, aromatase inhibitors; CDK4/6i, cyclin-dependent kinase 4/6 inhibitors.

**Table 2:** Univariate log-rank analysis of progression-free survival in patients with HR-positive advanced breast cancer treated with Dalpiciclib.

Characteristics	*n*	mPFS (Month)	95% CI	*p*-Value
Age (years)				
≥54	41	12.00	7.68–16.32	0.300
<54	35	11.00	7.40–14.70	
Menopausal status				
Yes	32	17.00	9.24–24.76	0.142
No	44	11.00	7.81–14.19	
ECOG score				
0–1	68	11.00	8.43–13.57	0.951
2	8	12.00	6.15–17.85	
Ki-67				
<30%	35	14.00	9.25–18.75	0.076
≥30%	41	10.00	6.75–13.25	
HR status				
ER + &PR−	12	10.00	5.56–14.45	0.782
ER + &PR+	64	12.00	9.90–14.10	
Percentage of ER				
<50%	6	10.00	NA	0.837
≥50%	70	12.00	10.09–13.91	
Percentage of PR				
<50%	39	12.00	6.06–17.94	0.411
≥50%	37	11.00	9.26–12.74	
HER2 status				
Negative	70	12.00	8.72–15.28	0.181
Positive	6	7.00	0.00–14.20	
Numbers of distant metastases				
<3	47	12.00	9.79–14.21	0.925
≥3	29	12.00	7.90–16.10	
Visceral metastasis				
Yes	30	12.00	10.65–13.36	0.614
No	46	10.00	6.79–13.21	
Only bone metastases				
Yes	13	16.00	2.74–29.26	0.480
No	63	11.00	9.15–12.85	
Liver metastasis				
Yes	24	10.00	7.25–12.75	0.733
No	52	12.00	10.14–13.86	
Line of dalpiciclib treatment				
First-line	30	17.00	9.19–24.81	<0.001
Second-line	35	10.00	7.31–12.67	
Third-line or later	11	4.00	1.84–6.16	
Dalpiciclib combined with endocrine drugs				
AI	47	12.00	7.16–16.84	0.027
Fulvestrant	29	8.00	2.58–13.42	
Prior lines of chemotherapy				
None	28	11.00	7.20–14.80	<0.001
First-line	27	24.00	NA	
Second-line	11	10.00	4.26–15.74	
Third-line or later	10	3.00	0.93–5.07	
Prior CDK4/6i treatment				
Yes	8	3.00	0.00–7.16	0.013
No	68	12.00	9.99–14.01	
Endocrine sensitivity				
Resistant	56	12.00	9.38–14.62	0.004
Sensitive	20	24.00	NA	

Abbreviations: mPFS, median progression-free survival; CI, confidence interval; ECOG, Eastern Cooperative Oncology Group; HR, hormone receptor; ER, estrogen receptor; PR, progesterone receptor; HER2, human epidermal growth factor receptor 2; AI, aromatase inhibitors; CDK4/6i, cyclin-dependent kinase 4/6 inhibitors; NA, not available.

**Table 4:** Safety outcomes for 51 patients with HR-positive advanced breast cancer treated with Dalpiciclib.

Adverse Events	Total	Grade 1	Grade 2	Grade 3	Grade 4
Haematological toxicities					
Neutropenia	37 (72.6%)	6 (11.8%)	10 (19.6%)	14 (27.5%)	7 (13.7%)
Leukopenia	39 (76.4%)	4 (7.8%)	15 (29.4%)	20 (39.2%)	0 (0%)
Anaemia	23 (45.1%)	14 (27.5%)	3 (5.9%)	4 (7.8%)	2 (3.9%)
Thrombocytopenia	11 (21.6%)	5 (9.8%)	3 (5.9%)	2 (3.9%)	1 (2.0%)
Non-haematological toxicities					
Aspartate aminotransferase increased	10 (19.6%)	10 (19.6%)			
Alanine aminotransferase increased	7 (13.7%)	7 (13.7%)			
Rash	3 (5.9%)	3 (5.9%)			
Increased bilirubin	5 (9.8%)	5 (9.8%)			
Blood creatinine increased	1 (2.0%)	1 (2.0%)			
Hyperuricaemia	2 (3.9%)	2 (3.9%)			
Sleep disorder	1 (2.0%)	1 (2.0%)			
Oral ulcer	1 (2.0%)	1 (2.0%)			
Edema	1 (2.0%)	1 (2.0%)			

## Data Availability

The data presented in this study is available on request from the corresponding author due to privacy and ethical restrictions.

## Supplementary Materials

The following supporting information can be downloaded at: https://www.mdpi.com/article/10.3390/cancers18061025/s1, Table S1: Comparison of baseline characteristics by line of dalpiciclib therapy; Table S2: Comparison of baseline characteristics between patients with and without complete adverse event data; Table S3: Sensitivity analysis using Cox regression model for patients who accepted first-line or second-line treatment of dalpiciclip.
