# Supplementary material for: Efficacy and Safety of Dalpiciclib in HR-Positive Advanced Breast Cancer: A Two-Center Retrospective Study
Source: Cancers (Basel). 2026 Mar 22;18(6):1025. doi: 10.3390/cancers18061025 (PMC13025841; doi:10.3390/cancers18061025)
Supplement: Supplementary file 1 [file cancers-18-01025-s001.zip › cancers-4126845-supplementary.pdf]

# Supplemental Materials: Efficacy and Safety of Dalpiciclib in HR-Positive Advanced Breast Cancer: A Two-Center Retrospective Study

**Jingjing Li [1,2,†], Zhiqiang Zong [2,†], Didi Zhu [2], Xiaojun Xu [3], Yunwen Yan [3], Jia Li [4], Fanfan Li [2,*] and Jiqing Hao [1,*]**

[1] Department of Oncology, The First Affiliated Hospital of Anhui Medical University, No. 218 Jixi Road, Hefei 230022, China
[2] Department of Oncology, The Second Affiliated Hospital of Anhui Medical University, No. 678 Furong Road, Hefei 230601, China
[3] Department of Breast Surgery, The First Affiliated Hospital of Anhui Medical University, No. 218 Jixi Road, Hefei 230022, China
[4] Department of Breast Surgery, The Second Affiliated Hospital of Anhui Medical University, No. 678 Furong Road, Hefei 230601, China
* Correspondence: efy124419@fy.ahmu.edu.cn (F.L.); haojiqing@ahmu.edu.cn (J.H.)
† These authors contributed equally to this work.

**Supplementary Table S1.** Comparison of baseline characteristics by line of dalpiciclib therapy.

| Characteristics | First-line (n = 30) | Second-line (n = 35) | Third-line or later (n = 11) | P-value |
|---|---|---|---|---|
| Age (years) | | | | |
| ≥ 54 | 16 (53.3) | 14 (40.0) | 5 (45.5) | 0.561 |
| < 54 | 14 (46.7) | 21 (60.0) | 6 (54.5) | |
| Menopausal status | | | | |
| Yes | 12 (40.0) | 16 (45.7) | 4 (36.4) | 0.823 |
| No | 18 (60.0) | 19 (54.3) | 7 (63.6) | |
| ECOG score | | | | |
| 0-1 | 29 (96.7) | 29 (82.9) | 10 (90.9) | 0.192 |
| 2 | 1 (3.3) | 6 (17.1) | 1 (9.1) | |
| Ki-67 | | | | |
| < 30% | 14 (46.7) | 18 (51.4) | 3 (27.3) | 0.373 |
| ≥ 30% | 16 (53.3) | 17 (48.6) | 8 (72.7) | |
| HR status | | | | |
| ER+&PR- | 4 (13.3) | 5 (14.3) | 3 (27.3) | 0.526 |
| ER+&PR+ | 26 (86.7) | 30 (85.7) | 8 (72.7) | |
| Percentage of ER | | | | |
| < 50% | 3 (10.0) | 2 (5.7) | 1 (9.1) | 0.805 |
| ≥ 50% | 27 (90.0) | 33 (94.3) | 10 (90.9) | |

| | | | | |
|---|---|---|---|---|
| Percentage of PR | | | | |
| < 50% | 15 (50.0) | 20 (57.1) | 7 (63.6) | 0.705 |
| ≥ 50% | 15 (50.0) | 15 (42.9) | 4 (36.4) | |
| HER2 status | | | | |
| Negative | 28 (93.3) | 33 (94.3) | 9 (81.8) | 0.388 |
| Positive | 2 (6.7) | 2 (5.7) | 2 (18.2) | |
| Number of distant metastases | | | | |
| < 3 | 21 (70.0) | 22 (62.9) | 4 (36.4) | 0.143 |
| ≥ 3 | 9 (30.0) | 13 (37.1) | 7 (63.6) | |
| Visceral metastasis | | | | |
| Yes | 17 (56.7) | 21 (60.0) | 8 (72.7) | 0.645 |
| No | 13 (43.3) | 14 (40.0) | 3 (27.3) | |
| Only bone metastases | | | | |
| Yes | 5 (16.7) | 6 (17.1) | 2 (18.2) | 0.993 |
| No | 25 (83.3) | 29 (82.9) | 9 (81.8) | |
| Liver metastases | | | | |
| Yes | 10 (41.7) | 10 (28.6) | 4 (36.4) | 0.858 |
| No | 20 (66.7) | 25 (71.4) | 7 (63.6) | |
| Dalpiciclib combined with endocrine drugs | | | | |
| Aromatase inhibitor | 27 (90.0) | 16 (45.7) | 4 (36.4) | < 0.001 |
| Fulvestrant | 3 (10.0) | 19 (54.3) | 7 (63.6) | |
| Prior lines of chemotherapy | | | | |
| None | 19 (63.3) | 8 (22.9) | 1 (9.1) | 0.003 |
| First-line | 8 (26.7) | 15 (42.9) | 4 (36.4) | |
| Second-line | 2 (6.7) | 7 (20.0) | 2 (18.2) | |
| Third-line or later | 1 (3.3) | 5 (14.3) | 4 (36.4) | |
| Prior CDK4/6 inhibitor use | | | | |
| Yes | 0 (0.0) | 5 (14.3) | 3 (27.3) | 0.026 |
| No | 30 (100.0) | 30 (85.7) | 8 (72.7) | |
| Endocrine sensitivity | | | | |
| Sensitive | 17 (56.7) | 2 (5.7) | 1 (9.1) | < 0.001 |
| Resistant | 13 (43.3) | 33 (94.3) | 10 (90.9) | |

Abbreviations: HR, hormone receptor; ER, estrogen receptor; PR, progesterone receptor; ECOG, Eastern Cooperative Oncology Group; CDK4/6, cyclin-dependent kinase 4/6.

**Supplementary Table S2.** Comparison of baseline characteristics between patients with and without complete adverse event data.

| Characteristics | Without adverse event data (n = 25) | With adverse event data data (n = 51) | P-value |
|---|---|---|---|
| Age (years) | | | |
| ≥ 54 | 15 (60.0) | 26 (51.0) | 0.459 |
| < 54 | 10 (40.0) | 25 (49.0) | |
| Menopausal status | | | |
| Yes | 15 (60.0) | 17 (33.3) | 0.027 |
| No | 10 (40.0) | 34 (66.7) | |
| ECOG score | | | |
| 0-1 | 22 (88.0) | 46 (90.2) | 0.769 |
| 2 | 3 (12.0) | 5 (9.8) | |
| Ki-67 | | | |
| < 30 | 8 (32.0) | 27 (52.9) | 0.085 |
| ≥ 30 | 17 (68.0) | 24 (47.1) | |
| HR status | | | |
| ER+&PR- | 6 (24.0) | 6 (11.8) | 0.169 |
| ER+&PR+ | 19 (76.0) | 45 (88.2) | |
| Percentage of ER | | | |
| < 50% | 2 (8.0) | 4 (7.8) | 0.981 |
| ≥ 50% | 23 (92.0) | 47 (92.2) | |
| Percentage of PR | | | |
| < 50% | 16 (64.0) | 26 (51.0) | 0.283 |
| ≥ 50% | 9 (36.0) | 25 (49.0) | |
| HER2 status | | | |
| Negative | 22 (88.0) | 48 (94.1) | 0.353 |
| Positive | 3 (12.0) | 3 (5.9) | |
| Number of distant metastases | | | |
| < 3 | 16 (64.0) | 31 (60.8) | 0.786 |
| ≥ 3 | 9 (36.0) | 20 (39.2) | |
| Visceral metastasis | | | |
| Yes | 11 (44.0) | 19 (37.3) | 0.572 |
| No | 14 (56.0) | 32 (62.7) | |
| Only bone metastases | | | |
| Yes | 3 (12.0) | 10 (19.6) | 0.408 |
| No | 22 (88.0) | 41 (80.4) | |
| Liver metastases | | | |
| Yes | 6 (24.0) | 18 (35.3) | 0.320 |
| No | 19 (76.0) | 33 (64.7) | |
| Number of Dalpiciclib treatment lines | | | |
| First-line | 10 (40.0) | 20 (39.2) | 0.953 |

| | | | |
|---|---|---|---|
| Second-line | 11 (44.0) | 24 (47.1) | |
| Third-line or later | 4 (16.0) | 7 (13.7) | |
| Dalpiciclib combined with endocrine drugs | | | |
| Aromatase inhibitor | 18 (72.0) | 29 (56.9) | 0.202 |
| Fulvestrant | 7 (28.0) | 22 (43.1) | |
| Number of prior lines of chemotherapy | | | |
| None | 10 (40.0) | 18 (35.3) | 0.960 |
| First-line | 9 (36.0) | 18 (35.3) | |
| Second-line | 3 (12.0) | 8 (15.7) | |
| Third-line or later | 3 (12.0) | 7 (13.7) | |
| Prior CDK4/6 inhibitor use | | | |
| Yes | 2 (8.0) | 6 (11.8) | 0.615 |
| No | 23 (92.0) | 45 (88.2) | |
| Endocrine sensitivity | | | |
| Sensitive | 6 (24.0) | 14 (27.5) | 0.748 |
| Resistant | 19 (76.0) | 37 (72.5) | |

Abbreviations: HR, hormone receptor; ER, estrogen receptor; PR, progesterone receptor; ECOG, Eastern Cooperative Oncology Group; CDK4/6, cyclin-dependent kinase 4/6.

**Supplementary Table S3.** Sensitivity analysis using Cox regression model for patients who accepted first-line or second-line treatment of dalpiciclip.

| Variable | Univariate analysis | | Multivariate analysis | |
|---|---|---|---|---|
| | HR (95%CI) | P-value | HR (95%Cl) | P-value |
| Line of dalpiciclib treatment | | | | |
| First-line | Reference | | Reference | |
| Second-line | 2.18 (1.10-4.32) | 0.026 | 4.16 (1.26-13.73) | 0.019 |
| Endocrine sensitivity | | | | |
| Sensitive | Reference | | Reference | |
| Resistant | 3.28 (1.36-7.92) | 0.008 | 1.61 (0.59-4.41) | 0.354 |
| Prior CDK4/6 inhibitor use | | | | |
| No | Reference | | Reference | |
| Yes | 4.49 (1.55-12.98) | 0.006 | 10.93 (2.40-49.79) | 0.002 |
| Dalpiciclib combined with endocrine drugs | | | | |
| Aromatase inhibitor | Reference | | Reference | |
| Fulvestrant | 1.48 (0.75-2.94) | 0.261 | 0.62 (0.23-1.71) | 0.357 |

Note: multivariate model was addtionally adjusted for age, HR status, HER2 status, and Ki-67. Abbreviations: PFS, progression-free survival; HR, hazard ratio; CI, confidence interval; HR status, hormone receptor status; HER2, human epidermal growth factor receptor 2. CDK4/6i, cyclin-dependent kinase 4/6 inhibitors; AI, aromatase inhibitors.
